# Chromium in Drinking Water: Association with Biomarkers of Exposure and Effect

**DOI:** 10.3390/ijerph111010125

**Published:** 2014-09-29

**Authors:** Eleni Sazakli, Cristina M. Villanueva, Manolis Kogevinas, Kyriakos Maltezis, Athanasia Mouzaki, Michalis Leotsinidis

**Affiliations:** 1 Lab of Public Health, Medical School, University of Patras, University Campus, Patras, GR 26504, Greece; E-Mail: elsazak@upatras.gr; 2 Centre for Research in Environmental Epidemiology (CREAL), Doctor Aiguader 88, Barcelona 08003, Spain; E-Mails: cvillanueva@creal.cat (C.M.V.); kogevinas@creal.cat (M.K.); 3 IMIM (Hospital del Mar Medical Research Institute), Barcelona 08003, Spain; 4 Universitat Pompeu Fabra (UPF), Barcelona 08002, Spain; 5 CIBER Epidemiología y Salud Pública (CIBERESP), Barcelona 08005, Spain; 6 National School of Public Health, Athens, GR 11521, Greece; 7 Health Centre of Aliartos, Aliartos, GR 32001, Greece; E-Mail: kyrmalt@gmail.com; 8 Division of Hematology, Department of Internal Medicine, Medical School, University of Patras, University Campus, Patras, GR 26504, Greece; E-Mail: mouzaki@upatras.gr

**Keywords:** chromium oral consumption, chromium hair, chromium blood, health effects, epidemiological study, biomarkers of exposure and effect

## Abstract

An epidemiological cross-sectional study was conducted in Greece to investigate health outcomes associated with long-term exposure to chromium via drinking water. The study population consisted of 304 participants. Socio-demographics, lifestyle, drinking water intake, dietary habits, occupational and medical history data were recorded through a personal interview. Physical examination and a motor test were carried out on the individuals. Total chromium concentrations were measured in blood and hair of the study subjects. Hematological, biochemical and inflammatory parameters were determined in blood. Chromium in drinking water ranged from <0.5 to 90 μg·L^−1^ in all samples but one (220 μg·L^−1^), with a median concentration of 21.2 μg·L^−1^. Chromium levels in blood (median 0.32 μg·L^−1^, range <0.18–0.92 μg·L^−1^) and hair (median 0.22 μg·g^−1^, range 0.03–1.26 μg·g^−1^) were found within “normal range” according to the literature. Personal lifetime chromium exposure dose via drinking water, calculated from the results of the water analyses and the questionnaire data, showed associations with blood and hair chromium levels and certain hematological and biochemical parameters. Groups of subjects whose hematological or biochemical parameters were outside the normal range were not correlated with chromium exposure dose, except for groups of subjects with high triglycerides or low sodium. Motor impairment score was not associated with exposure to chromium.

## 1. Introduction

Chromium is the 21st most abundant element in the Earth’s crust, with an overall concentration of 125 mg·kg^−1^ [1]. Although trivalent chromium (Cr(III)) is primarily of geological origin, hexavalent chromium (Cr(VI)) is derived mainly from industrial processes [2], but can also originate from the oxidation of naturally occurring Cr(III) by minerals containing Mn-oxides [3]. Physicochemical factors (pH, redox potential, *etc.*) control the speciation of chromium in the aquatic environment. The uniqueness of chromium stems from the totally different chemical behavior and toxicity of its two dominant forms. In food and dietary supplements, chromium is mainly Cr(III) and is considered to be essential for normal glucose, protein and fat metabolism, whereas in drinking water chromium is mainly Cr(VI).

Hexavalent chromium is a known human carcinogen by inhalation exposure in occupational settings [4]. However, when ingested, three competing processes dictate the fate of Cr(VI) in every single compartment of the human body: (a) transit through the compartment, (b) reduction to Cr(III) and (c) uptake/absorption into the tissues [5]. The physiological effects of these processes are subject to scientific debate. Some researchers argue that Cr(VI) is readily and sufficiently reduced by the saliva, the highly acidic gastric fluid, the bloodstream and the liver to Cr(III) [5,6,7], which cannot penetrate the cells due to its octahedral structure [2]. Others claim that an amount of Cr(VI) escapes reduction and can enter cells via the anionic transfer system [8,9]. Intracellular reduction of Cr(VI) generates intermediate Cr(V) and Cr(IV) ions and the final product Cr(III). Intermediate ions can produce reactive oxygen species (ROS) [10] and Cr(III) can react with DNA to form genotoxic DNA-adducts [11].

Health outcomes due to oral exposure to Cr(VI) have been reported in human and animal studies, and include alterations in hematological and biochemical parameters [12,13,14]. Decreased hemoglobin and hematocrit levels and increased total white blood cell counts have been described for an 18-year-old woman who ingested a few grams of potassium dichromate [15]. Microcytic and hypochromic anemia, characterized by decreased hematocrit, hemoglobin, mean corpuscular volume and mean corpuscular hemoglobin, was observed in rats and mice orally exposed to high concentrations (50–500 mg·L^−1^) of Cr(VI) compounds for 4 days to 1 year. Nevertheless, the animals appeared to be recovering from the anemia within 12 months [14]. Alterations in liver enzyme activities and serum components have been reported in humans after oral exposure to Cr(VI). Increased bilirubin and serum lactic dehydrogenase (LDH) were evident in a case of an accidental ingestion of a fluid containing 300 g·L^−1^ chromium trioxide. High levels of serum aspartate aminotransferase and alanine aminotransferase were observed in a boy 24 h after ingestion of 7.5 mg·kg^−1^ Cr(VI) [12]. In rats, increased activity of the same enzymes was observed, along with significant increases in serum triglyceride and glucose levels, and changes in the distribution and activity of alkaline phosphatase, acid phosphatase, glucose-6-phosphatase and cholinesterase [12]. Contradictory results have been reported regarding neurological effects of chromium. Diaz-Mayans *et al.* found a decrease in motor activity and balance in rats after administration of a daily dose of 240 μg chromium for 28 days [16], whereas Sutherland *et al.* [17] reported non-detectable chromium concentrations in the brain of rats after oral administration. As for human studies, there is one early report of abnormal deposits of chromium in three brains [18]. Decrease in the inflammation response has been reported among individuals exposed to chromium in a contaminated area [19].

Epidemiological studies evaluating the association between ingested Cr(VI) and adverse health effects in humans are relatively scarce. Two ecological studies conducted in China [20,21] and one in Greece [22], estimated cancer mortality (lung-, stomach-, *etc.*) associated with prolonged oral consumption of water contaminated with Cr(VI). These studies lacked individual exposure data [14,23]. The Zhang and Li study [20] provided sparse information about the magnitude and length of chromium exposure, and the Linos *et al.* study [22] has been questioned about the representativeness of the water samples considered, the lack of significant association between stomach cancer and chromium exposure [14], and possible exposure misclassification bias [24]. Two meta-analysis studies did not support an association between occupational exposure to Cr(VI) and gastrointestinal tract cancer mortality [25], or excess overall mortality [26]. A cross-sectional retrospective study in India evaluated health effects on population exposed to high concentrations of Cr(VI) that were far beyond the guideline value (approximately 20 mg·L^−1^) [27]. This study reported gastrointestinal and dermatological complaints and abnormal hematological function associated with living in communities with Cr(VI) polluted groundwater.

In Greece, there is an area in which drinking water was containing chromium at a concentration range of <0.5 to 90 μg·L^−1^, 80%–90% of which was Cr(VI). As a consequence, a large part of the population in this area has been exposed to chromium at concentrations exceeding the current drinking water guideline, which has been set to 50 μg·L^−1^ total chromium by the World Health Organization and the European Community. On July 2014, the California state of USA established a more stringent Maximum Contaminant Level of 10 μg·L^−1^ for Cr(VI) [28].

In order to investigate potential health effects in the general population associated with long-term consumption of drinking water in that area, an epidemiological cross-sectional survey was designed and implemented. Moreover, the chromium body burden was assessed through determination of chromium concentrations in blood and hair samples of the population. To our knowledge, this is the first epidemiological study that has been designed on personalized chromium exposure estimates.

## 2. Materials and Methods

### 2.1. Study Area

**Figure 1:**
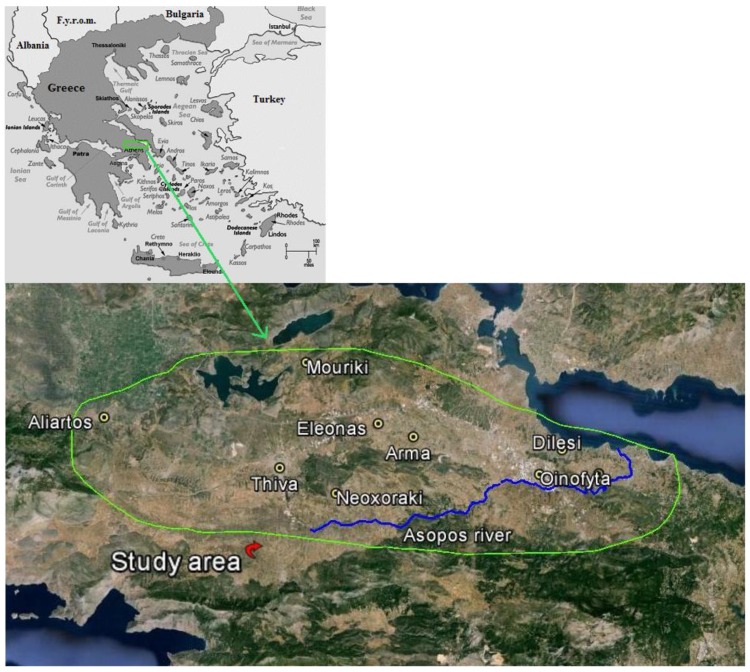
Map of the study area.

The study area is located at the catchment basin of Asopos river in Central Greece, about 60 km north of Athens (Figure 1). Both agricultural and industrial activities take place in this area. About 5%–15% of the national production of carrots, onions and potatoes occurs there [29,30]. The river, in its eastern part, runs through areas in which 13% of Greece’s industrial production takes place [29]. Mineralogical and geochemical studies support a geogenic origin for chromium in soils and groundwater of the area, mainly due to the Neogene and alluvial sediments and the serpentine soil [31]. The prevailing interchanging redox regimes (intermittently anoxic–oxic) in the presence of organic matter and other minerals (e.g., Mn oxides) favor the weathered Cr(III) to be transformed to Cr(VI), resulting in natural background concentrations up to 50 μg·L^−1^ [32,33]. However, unusually high Cr(VI) concentrations (up to 150 μg·L^−1^) in the groundwater of the region seem to have an additional anthropogenic origin from industrial activities [32]. The pollution of the river basin is believed to have started in 1969 when the industrialization of the area began and Asopos was proclaimed a “processed industrial waste receiver”.

The study area was divided into three sub-areas, namely A0, A1 and A2. Sub-area A1 (current exposure) consists of five villages in which the municipal water contains chromium because its source is the groundwater aquifer. Sub-area A2 (past exposure) consists of three villages and one town in which the origin of municipal water used to be local drills containing chromium until 2008 or 2009. Since then, the water supply has been diverted to receive surface, Cr-free water from Mornos reservoir, which also provides potable water to Athens. Sub-area A0 (reference) is a semi-urban place with traces of chromium in drinking water and similar socioeconomical and geographical characteristics with the rest of the study villages.

### 2.2. Environmental Samples Analyses

Water samples from the drinking water network (N = 50) in the study area were collected between October 2012 and February 2013. Chemical analyses regarding the parameters stated in the Drinking Water Directive (Council Directive 98/83/EC), including Cr(VI), were conducted according to the Standard Methods for the examination of water and wastewater [34] in the Laboratory of Public Health, which is accredited to ISO 17025. Total chromium was determined via graphite furnace atomic absorption spectrophotometry (GF-AAS), and Cr(VI) by a colorimetric method (APHA 3500-Cr B.). The detection limits were 0.5 μg·L^−1^ and 3 μg·L^−1^, respectively. Chromium results along with records of previous chromium determinations conducted in the Laboratory of Public Health were combined with data published by other researchers [35,36] and data provided by the municipalities. All combined data were produced either in accredited laboratories or via validated methods. A ten-year database (2004–2013) was constructed, with 676 determinations of total chromium and 572 determinations of Cr(VI) in municipal drinking water, including historical data of water sources for each study site.

All commercially available bottled water brands (N = 16) in the study area were examined for their chromium content. Three samples with different lot number per each brand were tested.

Crop samples (N = 48) were collected in the study area and analyzed for total chromium [37]. Briefly, approximately 0.5 g of dry sample was digested with 6 mL of 65% suprapure nitric acid in a microwave oven and diluted to a final volume of 10 mL. Chromium was determined by GF-AAS. Quantification was performed via standard solutions subjected to the same digestion procedure. The detection limit was found to be 0.002 μg·g^−1^ wet weight. Recovery of known chromium amounts added to the crop samples before wet digestion varied from 95 to 102%. The analyses results were combined with previously published data of chromium content in local crops [37,38]. Overall, 273 determinations of chromium in local crops, conducted either in accredited laboratories or via validated methods, were available.

### 2.3. Study Population

Eligible participants were men and women, aged 25–69 years, who were residents of the study area for at least the last seven consecutive years. This way, an exposure window from two to 69 years was covered. The size of the study population was calculated through statistical power scenarios to be about 300 individuals, with 40% from area A1 (current exposure), 40% from area A2 (past exposure) and 20% from area A0 (reference area). A stratified random sampling method was employed, by dividing the sampling population in non-overlapping sex and age strata, to obtain a representative sample. Potential study subjects were selected randomly from local telephone directories. A first phone contact took place to explain the aims of the study to potential participants, verify that they fulfilled the inclusion criteria, and invite them to participate. The response rate was 82% and 304 subjects were recruited.

After accepting to participate, it was arranged for the study subjects to visit the local health center of each village by appointment. There, the subjects met with a general practitioner and a health visitor, who were members of the research team, and signed a written informed consent. The study was approved by the Ethics Committee of the University of Patras (Re: 30/20-12-2012). The recommendations outlined in the Declaration of Helsinki [39] have been followed.

### 2.4. Interview and Medical Examination

Study subjects were recruited from February to December 2013 for a personal interview. A questionnaire was administered, a medical examination was conducted and biological samples (hair, blood) were collected.

(A) *Questionnaire.* A questionnaire was designed and tested in a pilot population of 30 individuals. The practitioner filled in the questionnaire through personal interview to collect information on: demographic characteristics (age, sex, residential history, marital status, educational level, economical status, working status); medical history (morbidities, hereditary diseases); lifestyle (physical activity, smoking, coffee and alcohol consumption); source of drinking water supply (municipal/bottled/private well/other) currently and in the past; daily usual water consumption; occupational history; use of antacids and proton-pump inhibitors (PPIs); weekly consumption frequencies of major food groups, *i.e.*, meat, fish, vegetables, legumes, dairy products, sweets and fruits, assessed by a 7-day food frequency questionnaire. A specific question concerning consumption of local crops was also included. Medical history included the following diagnosed diseases: Heart trouble, hypertension, osteoarthritis/arthritis/rheumatism, cancer/leukemia/lymphoma, asthma, emphysema/chronic bronchitis, diabetes, cataract, stroke, broken/fractured bone, chronic nervous/emotional problems, Parkinson’s disease, hypercholesterolemia/hypertriglyceridemia, circulatory problems, migraines/headaches and thyroid disturbances. The section “occupational history” included a list of occupations with potential exposures to chromium according to IARC [1]. Moreover, data about the worksite (production line or office), the time (currently or in the past) and the duration of occupation exposure were recorded for each recruit.

(B) *Physical examination.* Weight and height, systolic and diastolic blood pressure were recorded for each subject.

(C) *Neurological tests.* The practitioner conducted the Unified Parkinson’s Disease Rating Scale (UPDRS)—Motor examination test, which is composed of 14 questions and assesses motor disability and impairment.

### 2.5. Biomarkers of Exposure and Effect; Collection and Analysis of Blood and Hair Samples

Concentrations of chromium in blood and hair samples of the participants were considered as biomarkers of exposure. Hematological, biochemical, inflammatory parameters and the neurological score were considered as biomarkers of effect.

Approximately 12–15 mL of peripheral blood was drawn from each individual by the health visitor and transferred into BD Vacutainer plus EDTA K3 tubes (for whole blood analysis) and BD Vacutainer Plus S.S.T. tubes (for serum analysis after centrifugation). The samples were placed into isothermal boxes (4 °C) and were transferred to the corresponding laboratories for analysis. Full blood count was conducted in whole blood within 12 h after sampling. Biochemical analysis and cytokine concentrations were determined in serum (within 12 h after sampling). Total chromium was analyzed in whole blood according to McAughey and Smith [40] with a modification: 0.5 mL whole blood was rigorously mixed with 2 mL of 0.2% HNO_3_ and 0.1% TritonX-100, and the total chromium concentration was determined by Electrothermal Atomic Absorption Spectrometry (Shimadzu AA-6300, Shimadzu GFA-Ex7i graphite furnace and Shimadzu ASC-6100 auto-sampler). ClinChek^®^ whole blood controls, purchased by RECIPE and subjected to the same procedure with blood samples, were used to construct calibration curves with final chromium concentrations 0.34, 1.21 and 2.38 μg·L^−1^. Quality-control samples were prepared by spiking aliquots of whole blood with aqueous chromium standards at three concentration levels: 0.20, 0.40 and 0.80 μg·L^−1^. The uncertainty of the method was estimated to be U = 0.09 × C − 0.07. Under the experimental conditions used, the limit of detection was 0.18 μg·L^−1^. Determination of cytokine concentrations was performed in a subset of 79 subjects, on a BD FACSArray Bioanalyzer using the Cytometric Bead Array (CBA) assay (Human Inflammation Kit, BD Biosciences, San Diego, CA, USA).

Samples of head hair (0.5–1 g) were cut from the scalp of the occipital region and up to a distance of 2 cm. The health visitor collected the samples using stainless steel scissors and placed them in metal-free plastic containers. Hair samples were treated according to Leotsinidis and Kondakis [41], with modifications: immersion in acetone for 20 min, a rinse in MilliQ water, thorough washing with 0.1% SDS solution in an ultrasonic bath for 15 min, followed by washing with MilliQ water in the bath for 15 more min, filtration and overnight drying at 60 °C. The samples were subjected to a microwave-assisted closed wet digestion (approximately 0.2 g sample and 7 mL supra-pure HNO_3_). Standard curves were obtained using standard solutions subjected to the same digestion procedure. The detection limit was found to be 0.03 μg·g^−1^. Precision was estimated at 5% by replicate measurements on 30 hair samples. Recovery of known chromium amounts added to the hair samples before wet digestion varied from 92% to 104%.

### 2.6. Estimates of Lifetime Chromium Exposure Dose

The medians of chromium concentrations were calculated for each calendar year, village/town of residence and drinking water source, using the constructed database (term C_i_ in equation (1)). Annual chromium exposure dose through drinking water was calculated for each individual according to the Water Ingestion Exposure Dose equation [42] by applying year-, village-, and drinking water source-specific chromium concentrations to each year based on the residential and drinking water histories:

D = (C_i_ × IR × EF)/BW (1)

D = annual chromium exposure dose (μg·kg^−1^ BW year^−1^);

C_i_ = chromium concentration in drinking water in village_i_ or in bottled water_i_ (μg·L^−1^);

IR = intake rate of water for the individual (L·year^−1^);

EF = exposure factor (unitless);

BW = body weight (kg).

Intake rate (L·year^−1^) was calculated by multiplying the daily consumption of drinking water (L·d^−1^) captured by the questionnaire, times the days per year (365 d·year^−1^). Exposure factor was equal to 1, since average yearly consumption was estimated. Lifetime chromium exposure dose from water (hereinafter “exposure dose”) (μg·kg^−1^ BW) was finally calculated by adding the annual chromium exposure doses (D) for all living years per each individual.

### 2.7 Statistical Analysis

Statistical analysis was performed by IBM SPSS v.21 statistical software (SPSS Inc., Chicago, IL, USA). Continuous variables are presented by range and median and categorical variables as frequencies. Non-parametric Kruskal-Wallis *H* test was employed to compare groups, followed by Mann-Whitney U test. Differences in proportions were tested using the Pearson chi square test. Spearman’s rho correlation coefficient was calculated to assess bivariate associations. Linear regression models were constructed, after log-transformations if needed, to describe the associations between (i) exposure dose and chromium in blood or hair, (ii) exposure dose and the hematological/biochemical parameters, inflammatory factors and other outcomes and (iii) chromium body burden (Cr in blood/Cr in hair) and the hematological/biochemical parameters, inflammatory factors and other health outcomes. Multivariate logistic regression models were employed to investigate associations between (i) either exposure dose or blood or hair chromium levels with self-reported diseases, diagnosed by a physician, (ii) exposure dose and groups of subjects with normal and “out of normal” values in certain hematological/biochemical parameters. The statistical significance level was set at a = 0.050.

## 3. Results

### 3.1. Chromium in Environmental Samples

The results of chromium determinations in environmental samples are presented in Table 1. Levels in drinking water ranged from non-detectable (<0.5 μg·L^−1^) to 220 μg·L^−1^ Cr. The 220 μg·L^−1^ value was observed only in one sample, whereas the next lower concentration was 90 μg·L^−1^. The Cr(VI) to total Cr ratio was 0.8–0.9. Annual variation was minimal in each village (Kruskal-Wallis test, *p* > 0.050). Bottled water brands were classified into two groups as for their chromium content, one (group A) with negligible chromium and the other (group B) with a chromium range of 19.0–24.0 μg·L^−1^ (Table 1). High inter-crop variability was observed, whereas intra-crop variability was not significant (Table 1).

### 3.2 Characteristics of the Study Population

The characteristics of the study population are shown in Table 2. Most characteristics did not differ across the three areas. Differences were found in educational level (χ^2^, *p* < 0.001), chromium occupational exposure (χ^2^, *p* = 0.046) and physical activity (χ^2^, *p* = 0.039). To note, 42% of the people in A2 area have been drinking municipal water, whereas the corresponding percentage in A0 area is 82%. 

**Table 1:** Chromium concentrations in environmental samples.

Sample	*N*	Median	Range	25th Percentile	75th Percentile
**Drinking water, Total chromium (μg·L^−1^ Cr)**					
Area A1 ^a^ (2005–2013)	*290*	25.0	0.7–39.9	13.0	28.7
Area A2 (2004–2008/09 ^b^)	*284*	21.0	<0.5–220.0	16.2	29.7
Area A2 (2008/09 ^b^–2013)	*70*	0.5	<0.5–3.2	<0.5	0.7
Area A0 (2005–2013)	*32*	1.1	<0.5–3.5	0.7	1.6
Total	*676*	21.2	<0.5–220.0	11.7	28.0
**Drinking water, Hexavalent chromium (μg·L^−1^ Cr^+6^)**					
Area A1 (2005–2013)	*270*	23	<3–38	12	26
Area A2 (2004–2008/09 ^b^)	*231*	16	<3–196	12	22
Area A2 (2008/09 ^b^–2013)	*50*	<3	<3	<3	<3
Area A0 (2005–2013)	*21*	<3	<3	<3	<3
Total	*572*	17	<3–196	9	24
**Bottled water, Total chromium (μg·L^−1^ Cr)**					
Group A brands	*30*	<0.5	<0.5–3.0	<0.5	<0.5
Group B brands	*18*	21.0	19.0–24.0	19.0	22.0
**Crops, Total chromium (μg·g^−1^ wet weight)**					
Carrots	*71*	0.053	0.003–0.240	0.033	0.071
Potatoes	*65*	0.039	0.002–1.100	0.027	0.120
Onion and garlic	*110*	0.026	0.002–1.200	0.007	0.073
Cabbage	*2*	0.010	0.006–0.013	0.006	0.013
Fresh leafy vegetables	*10*	0.028	0.007–0.099	0.026	0.050
Other fresh vegetables	*15*	0.021	0.005–0.133	0.010	0.033

Notes: **^a^** Area A1: current Cr exposure area, Area A2: past Cr exposure area, Area A0: reference area; **^b^** 2008 or 2009, depending on the supply change in individual villages.

**Table 2:** Description of the study population.

		Area Classification ^a^			
		A1 *(N = 122)*	A2 *(N = 115)*	A0 *(N = 67)*	Total *(N = 304)*
**Sex (%)**	Men	50.8	47.0	46.3	48.4
	Women	49.2	53.0	53.7	51.6
**Education level (%)**	Illiterate/primary	27.9	18.3	14.9	21.4
	Secondary/high	51.6	47.0	41.8	47.7
	Technological/university	19.7	34.8	43.3	30.6
	Not Answered	0.8	0.0	0.0	0.3
**Marital status (%)**	Single	18.9	13.0	17.9	16.4
	Married	74.6	75.7	71.6	74.3
	Separated/divorced/widowed	6.6	11.3	10.4	9.2
**Occupational status (%)**	Work force	64.8	74.8	67.2	69.1
	Unemployed	9.0	7.0	13.4	9.2
	Household	18.9	7.0	10.4	12.5
	Retired	7.4	11.3	9.0	9.2
**Occupational exposure to Cr (%)**	Current work in production in factories with Cr exposure	4.9	0.9	3.0	3.0
	Past work in production in factories with Cr exposure	22.1	21.7	19.4	21.4
	Current/past work in office in factories with Cr exposure	3.3	13.9	4.5	7.6
	Other no occupational exposure	69.7	63.5	73.1	68.1
**Income (€) (%)**	<9000	18.9	16.5	22.4	18.8
	9000–16000	36.1	26.1	34.3	31.9
	16000–24000	24.6	27.0	16.4	23.7
	>24000	9.0	23.5	19.4	16.8
	Not Answered	11.5	7.0	7.5	8.9
**Smoking (%)**	Current	43.4	43.5	49.3	44.7
	Ex	13.9	27.0	10.4	18.1
**Coffee consumption (%)**	Yes	92.6	88.7	94.0	91.4
**Alcohol consumption (%)**	Yes	64.8	57.4	58.2	60.5
**Physical activity (%)**	Yes	43.4	55.7	61.2	52.0
**Self-reported health status (%)**	Very good	14.8	27.0	26.9	22.0
	Good	59.0	51.3	44.8	53.0
	Neither good, nor bad	22.1	18.3	25.4	21.4
	Bad/very bad	3.3	3.5	1.5	3.0
	Not Answered	0.8	0.0	1.5	0.7
**Diagnosed diseases (%)**	Yes	84.4	81.7	85.1	83.6
**Antacids or Proton-pump inhibitors (PPIs) (%)**	Yes	19.7	17.4	13.4	17.4
**Type of drinking water (%)**	Municipal supply	64.8	41.7	82.1	59.9
	Bottled water	18.0	40.9	3.0	23.4
	Both	17.2	17.4	14.9	16.8
**Consumption oflocal crops (%)**	Exclusively	22.3	7.0	1.5	11.8
	Usually	76.9	80.9	97.0	82.6
	Seldom/never	0.0	7.8	0.0	3.0
	Not Answered	0.8	4.3	1.5	2.6
**Age (years)**	Median	50	48	47	48
	*Range*	*25–69*	*29–66*	*30–63*	*25–69*
***Lifetime chromium exposure dose via drinking water***					
**Exposure dose (μg·kg^−1^ BW)**	Median	3738.0	1654.6	307.1	1533.1
	*Range*	*26.1–21574.7*	*8.6–29281.1*	*54.0–3736.7*	*8.6–29281.1*

Notes: **^a^**: Area A1: current Cr exposure area, Area A2: past Cr exposure area, Area A0: reference area.

The attitude towards local crops’ consumption is similar: 22% of subjects in A1 area reported to consume local crops exclusively whereas the corresponding percentage for A2 area was only 7%. To note, 34% of the population in A1 area are farmers and consume their own products. The calculated exposure doses were significantly different among the three areas (Kruskal Wallis test, *p* < 0.001), with the lowest found in A0 area, as it was expected.

### 3.3 Chromium in Blood and Hair Samples

**Figure 2:**
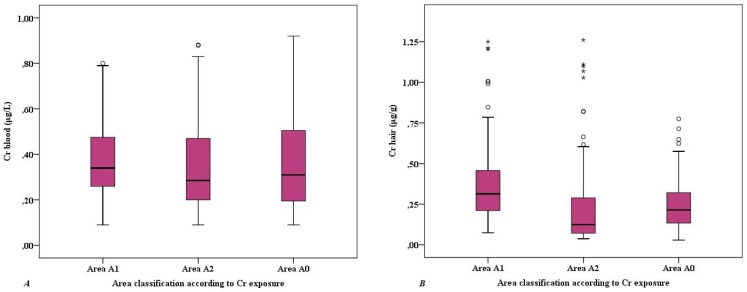
Chromium concentrations in blood (A) and hair (B) samples of the subjects in the three areas.

Chromium concentrations in blood and hair samples are presented in Figure 2. Blood levels did not differ among the three areas (Kruskal Wallis test, *p* = 0.130). The overall median was 0.32 μg·L^−1^ with a range <0.18–0.92 μg·L^−1^. In hair samples, the median concentration was 0.22 μg·g^−1^, with a range from 0.03 to 1.26 μg·g^−1^. Chromium in hair differed among the three areas (Figure 2). Chromium levels in blood and in hair were weakly correlated (Spearman’s rho 0.291, *p* < 0.001).

Table 3 presents the linear regression models between exposure dose and chromium levels in blood or in hair. Variables tested were age, sex, marital status, income, educational level, smoking pack-years, alcohol and coffee consumption, self-reported health status, diagnosed diseases, physical activity, occupational chromium exposure, use of antacids or PPIs, shower frequency, food frequency consumption and consumption of local crops.

**Table 3:** Association between exposure dose and chromium levels in hair or in blood.

	Model ^a^			
	Cr in blood *(N = 300)*		Cr in hair (ℓn) *(N = 299)*	
	R^2^ = 0.135, (*p < 0.001*)		R^2^ = 0.184, (*p < 0.001*)	
*Parameter*	*SRC ^b^*	*p value*	*SRC*	*p value*
Exposure dose (ℓn)	**0.134**	*0.023*	**0.226**	*<0.001*
Age	−0.025	*0.687*	−0.102	*0.078*
Sex (women)	0.023	*0.714*	−0.091	*0.124*

Notes: **^a^** Regression models adjusted for: age, sex, Cr occupational exposure, consumption of local crops and frequencies of certain food items’ consumption; **^b^** SRC: standardized coefficient.

The final models were adjusted for age, sex, occupational chromium exposure, consumption of local crops and weekly frequency of certain food items. In the blood final model, a significant, yet weak association between exposure dose and chromium blood levels was observed. Adjusting variables did not gain statistical significance (data not shown).

A positive association was also observed between exposure dose and chromium hair levels. Other associations that were found in the adjusted hair model but are not presented in Table 3 are: Occupational exposure to chromium, specifically the current workers in the production line of factories with chromium exposure (SRC = 0.119, *p* = 0.032) and consumption of certain food items, namely legumes (SRC = 0.127, *p* = 0.021), cereals (SRC=0.145, *p* = 0.009) and vegetable oils (SRC = −0.136, *p* = 0.016). Consumption of local crops (“usually” *versus* “exclusively”) was correlated negatively with chromium levels in hair (SRC = −0.146, *p* = 0.022).

### 3.4 Exposure Dose and Hematological/Biochemical and Other Outcomes

Table 4 demonstrates the associations between exposure dose with hematological and biochemical parameters, as revealed by the multivariate adjusted models. The highest R^2^ for hematological parameters was achieved in the multivariate model of exposure dose and hemoglobin (R^2^ = 0.450), and for biochemical parameters of exposure dose and albumin (R^2^ = 0.230). Exposure dose was positively associated with systolic and diastolic blood pressure and with interleukin-12p70. The score of the motor examination test was not associated with exposure dose.

### 3.5 Blood/Hair Chromium Levels and Hematological/Biochemical and Other Outcomes

Chromium levels in blood presented positive weak associations with certain biochemical parameters (Table 4). The highest R^2^ obtained in these models was 0.171 for γGT and the highest SRC was 0.248 for ℓn(glucose). No association was found between chromium in blood and hematological parameters, systolic and diastolic pressure, inflammation factors and motor impairment.

The associations between chromium hair concentrations and health outcomes are also presented in Table 4. The highest R^2^ obtained in these models was 0.378 for hematocrit and 0.182 for HDL. Chromium body burden, as expressed by chromium concentrations in hair, showed no association with other outcomes (blood pressure, motor impairment and inflammation activity). All models were adjusted for sex and age.

### 3.6 Chromium and Self-Reported Health Outcomes

No significant associations were found between self-reported diseases, diagnosed by a physician, with either exposure dose or blood or hair chromium levels. The models were adjusted for sex and age.

### 3.7 Exposure Dose and Groups of Subjects with “Out of the Normal” Values

To investigate further whether the associations that were found between the exposure dose and the hematological/biochemical parameters reflect alterations, subjects were classified into groups according to normal or “out of normal” values per each parameter. These cut-off values, which were given by the biochemical laboratory where the analyses were conducted, along with details about the population distribution can be found in the supplementary section (Table S1). Multivariate logistic regression models that kept exposure dose (*p* < 0.050) were only those regarding triglycerides and sodium (data not shown). The corresponding percentages of “out of the normal” groups were 20.2% and 6.0% of the total population. The hypertensive group (systolic ≥140 mmHg and/or diastolic blood pressure ≥90 mmHg), which constituted 38.7% of the subjects, showed a trend of association with exposure dose, though at the border of statistical significance (*p* = 0.066).

**Table 4:** Association of exposure dose, chromium in blood, chromium in hair with hematological/biochemical and other outcomes.

	Exposure Dose (ℓn) ^a^		Cr in Blood ^b^		Cr in Hair (ℓn) ^b^	
	*SRC ^c^*	*p*	*SRC*	*p*	*SRC*	*p*
**Hematological parameters *(N = 296)***						
Red Blood Cells (10^12^/L)	0.007	*0.896*	−0.085	*0.111*	−0.091	*0.098*
Hemoglobin (g/dL)	**−0.093**	*0.041*	−0.038	*0.396*	−0.059	*0.210*
Hematocrit (%)	**−0.094**	*0.048*	−0.061	*0.192*	**−0.099**	*0.041*
Mean Corpuscular Volume (fL)	−0.098	*0.088*	0.035	*0.550*	−0.002	*0.969*
Mean Corpuscular Hemoglobin (pg)	−0.101	*0.083*	0.060	*0.304*	0.040	*0.502*
Mean Cell Hemoglobin Concentration (g/dL)	−0.001	*0.990*	0.084	*0.154*	**0.173**	*0.004*
Red blood cell Distribution Width (%)	0.072	*0.205*	−0.077	*0.169*	**−0.191**	*0.001*
White Blood Cells (10^9^/L)	−0.032	*0.587*	0.025	*0.670*	0.069	*0.255*
Granulocytes percentage (%)	−0.081	*0.177*	−0.082	*0.167*	−0.063	*0.297*
Lymphocytes percentage (%)	0.070	*0.243*	0.071	*0.228*	0.054	*0.372*
Mid Cells percentage (%)	0.081	*0.161*	0.093	*0.110*	0.041	*0.494*
Granulocytes total count (10^9^/L)	−0.014	*0.811*	0.023	*0.698*	0.032	*0.599*
Lymphocytes total count (10^9^/L)	0.023	*0.705*	0.084	*0.155*	0.111	*0.067*
Mid Cells total count (10^9^/L)	0.097	*0.093*	0.043	*0.465*	0.073	*0.221*
Platelets (10^9^/L)	0.012	*0.843*	0.052	*0.364*	**0.202**	*<0.001*
Plateletcrit (%)	0.020	*0.719*	0.081	*0.148*	**0.229**	*<0.001*
Mean Platelet Volume (fL)	0.050	*0.412*	−0.023	*0.695*	0.020	*0.744*
Platelet Distribution Width (fL)	0.095	*0.119*	−0.020	*0.736*	0.005	*0.930*
Large Platelet Concentration Ratio (%)	0.082	*0.168*	0.019	*0.757*	−0.013	*0.834*
**Biochemical parameters *(N = 304)***						
ℓn(Glucose) (mg/dL)	0.107	*0.054*	**0.248**	*0.000*	**0.193**	*0.001*
Urea (mg/dL)	−0.049	*0.389*	**−0.143**	*0.012*	−0.011	*0.855*
Creatinine (mg/dL)	−0.011	*0.830*	0.017	*0.729*	−0.026	*0.611*
Uric acid (mg/dL)	0.048	*0.308*	0.044	*0.362*	0.074	*0.133*
Cholesterol (mg/dL)	0.021	*0.723*	0.096	*0.103*	**0.268**	*<0.001*
ℓn(Triglycerides) (mg/dL)	**0.144**	*0.009*	0.047	*0.402*	0.102	*0.076*
High-Density Lipoprotein (mg/dL)	**−0.113**	*0.034*	−0.012	*0.816*	**0.119**	*0.030*
Low-Density Lipoprotein (mg/dL)	0.003	*0.963*	0.101	*0.086*	**0.216**	*<0.001*
Potassium (mmol/L)	0.049	*0.392*	**0.120**	*0.038*	**0.127**	*0.033*
Sodium (mmol/L)	**−0.145**	*0.011*	−0.099	*0.088*	**−0.142**	*0.018*
Alkaline phosphatase (U/L)	**0.120**	*0.035*	**0.216**	*0.000*	**0.131**	*0.026*
Aspartate aminotransferase (U/L)	0.040	*0.476*	0.035	*0.534*	0.029	*0.609*
ℓn(Alanine aminotransferase) (U/L)	−0.027	*0.600*	0.053	*0.334*	0.032	*0.568*
γ-Glutamyl Transpeptidase (U/L)	0.055	*0.294*	**0.109**	*0.041*	−0.024	*0.658*
Creatine Kinase (U/L)	0.000	*0.998*	0.026	*0.651*	0.048	*0.412*
Lactate Dehydrogenase (U/L)	0.101	*0.074*	**0.123**	*0.033*	**0.175**	*0.003*
Amylase (U/L)	**0.159**	*0.005*	**0.177**	*0.002*	**0.262**	*<0.001*
Bilirubin total (mg/dL)	−0.111	*0.055*	−0.068	*0.243*	−0.029	*0.627*
Bilirubin direct (mg/dL)	−0.095	*0.103*	−0.014	*0.809*	0.016	*0.782*
Bilirubin indirect (mg/dL)	−0.077	*0.185*	−0.110	*0.059*	−0.088	*0.141*
Albumin (g/dL)	**0.213**	*<0.001*	**0.232**	*0.000*	**0.164**	*0.005*
Proteins total (g/dL)	**0.144**	*0.012*	**0.237**	*0.000*	**0.376**	*<0.001*
Sfairines	−0.094	*0.108*	−0.019	*0.748*	**0.184**	*0.002*
Calcium (mg/dL)	**0.117**	*0.044*	**0.219**	*0.000*	**0.321**	*<0.001*
Phosphate (mg/dL)	0.098	*0.089*	0.101	*0.079*	**0.121**	*0.041*
C-reactive Protein (mg/dL)	0.062	*0.276*	0.032	*0.587*	−0.042	*0.479*
**Inflammation response *(N = 79)***						
ℓn(IL-6)	−0.097	*0.389*	−0.014	*0.899*	−0.133	*0.248*
ℓn(IL-8)	−0.050	*0.674*	0.149	*0.196*	−0.095	*0.422*
ℓn(IL-10)	0.022	*0.862*	−0.022	*0.846*	−0.173	*0.135*
ℓn(IL-12)	**0.308**	*0.011*	−0.131	*0.262*	0.022	*0.855*
**Other outcomes *(N = 300)***						
Diastolic pressure	**0.116**	*0.042*	0.061	*0.288*	0.073	*0.217*
Systolic pressure	**0.142**	*0.010*	0.033	*0.547*	0.107	*0.057*
ℓn(neurologic score)	−0.002	*0.972*	0.009	*0.866*	0.024	*0.667*

Notes: **^a^** Regression models adjusted for: age, sex, Cr occupational exposure, smoking, alcohol consumption, physical activity, consumption of local crops and frequencies of food items’ consumption; **^b^** Regression models adjusted for: age and sex; **^c^** SRC: Standardized coefficients.

## 4. Discussion

The present epidemiological study reports slight associations between exposure dose and certain hematological and biochemical parameters. However, most of these associations have not been expressed as deviations from normal range. No motor impairment was observed in the exposed subjects. Even though positive associations were found between exposure dose and chromium levels in blood and hair, no deviation from “normal chromium concentration” was evident in subjects exposed to up to 90 μg·L^−1^ chromium via drinking water for a long time period. Occupational exposure to chromium seems to play a role in chromium hair levels but only for individuals currently working in the production line of factories with chromium exposure compared to other occupational categories. Past factory workers or current workers in factory departments other than the production line did not show such an association.

The study population has being exposed to chromium via the oral route both by drinking water ingestion and local crops consumption. Exposure dose (i.e. the lifetime chromium exposure dose from water) was calculated for each individual, based on his residence, quantity and source of drinking water. In that way, exposure misclassification was eliminated. It should be mentioned that almost all chromium in drinking water is in the hexavalent form (Table 1), in agreement with the EFSA report [43]. Total chromium was considered in the calculations, since the use of different oxidizing agents and doses for tap water treatment leads to slight variations (0.8–0.9) in the fractions of Cr(VI) [43]. The study population has not been exposed to other toxicants through drinking water, since all other chemical parameters tested in the water samples were found within the current guideline values. The water analyses results are presented in the supplementary section (Table S2). A personal chromium exposure dose from food could not be calculated because there are no data for chromium concentrations in all food items, either local crops or purchased food. Taking into account that the crop samples were representative of the whole study area and no intra-crop variability was observed in chromium concentrations, the frequency of the weekly consumption of food items for each individual was used as the explanatory variable for chromium exposure through food consumption.

Chromium in body fluids (e.g., blood and urine) is the biomarker of choice used to either identify or quantify exposure to chromium [12]. To assess the determined metal concentrations in body fluids and tissues, comparison with reference or normal concentration values based on measurements on unexposed healthy subjects is needed [44]. In the present study, chromium blood concentrations in all subjects fell within the “normal range” published in the literature (<0.04–1.20 μg·L^−1^) [45,46]. Blood chromium concentrations as high as 151.65 μg·L^−1^ have been reported in occupationally exposed welders in India [47]. Human hair is a reliable and convenient biological indicator of present and past exposure and can reflect long-term exposure to metals (unlike blood or urine) [48]. In the literature, large variations in normal metal hair concentrations are common, attributed to factors such as dietary habits, lifestyle, geochemical environment, age, sex, hair color and smoking habits [48]. Review articles report reference values for chromium in hair from 0.03‒1.20 μg·g^−1^ [49] and 0.001‒4.56 μg·g^−1^ [50]. In a previous Greek study, the 25th and 75th percentiles of chromium in hair were found 0.33 μg·g^−1^ and 0.84 μg·g^−1^, respectively [41]. Chromium concentrations in our population fell within or below the reported reference values. In comparison, in tannery workers chromium hair concentrations were found two orders of magnitude higher (17 μg·g^−1^) [51]. The “normal” values found in all subjects indicate that the chromium body burden has remained at low levels. The even lower chromium hair concentrations found in A2 area in our study may be attributed not only to the cessation of chromium exposure via drinking water during the last 4 years, but also to the emphasis given by the local media to the topic of environmental chromium pollution in that region, that has influenced the dietary habits and lifestyle of the local population.

In our population, weak negative associations were found in the models between exposure dose and hematocrit or hemoglobin, in agreement with previous studies [12]. However, groups of subjects with lower than the normal values were not correlated with exposure dose, as shown by the logistic models. Consequently, subjects have not expressed symptomatic alterations in these hematological parameters, in spite of their long-term exposure. Regarding biochemical parameters, positive weak associations of chromium ingestion with albumin, total proteins and triglycerides found in our study are in agreement with previously reported increases in rats after orally administered Cr(VI) [13,52]. On the contrary, others reported decrease in the amounts of triglycerides [14] and inhibition of the alkaline phosphatase activity [13]. The negative association between exposure dose and sodium has not been reported previously, whereas no associations were observed for transaminases, in disagreement with previous studies [12]. Of the study subjects deviating from normal range, only the groups with high triglycerides or low sodium were associated with exposure dose.

In our study, by measuring an array of cytokines involved in inflammation in blood, we found a positive correlation between interleukin-12 (IL-12) levels and chromium exposure dose, whereas the levels of other cytokines measured (IL-6, IL-8 and IL-10) were not elevated and did not yield significant correlations with chromium exposure. Snyder *et al.* [19] found a significant (36%) decrease in the levels of interleukin-6 (IL-6) in individuals exposed to chromium, but no such association was found in our study. These results are interesting, because IL-12 is produced by activated antigen-presenting cells, mainly macrophages and dendritic cells, and promotes cell-mediated immunity. IL-12 stimulates production of interferon-γ from T-cells and natural-killer cells, and is essential for the induction of pro-inflammatory Th1 responses that are mainly required for protection against intracellular pathogens [53]. In general terms, this can be considered as a beneficial effect; on the other hand, maintaining a chronic subclinical inflammatory response can lead, long-term, to inflammation-linked pathological conditions, such as tumorigenesis. To counter-argue, the low levels of IL-6, IL-8 and IL-10 in the sera of the study subjects are indicative of a lack of a generalized inflammatory response. It would be, therefore, interesting to investigate whether feedback control mechanisms operate in parallel, to limit the damage caused by IL-12-driven inflammation stress, long-term.

Blood pressure was positively correlated with exposure dose. However, only a trend of association was observed between hypertensive subjects and exposure dose, as shown by the adjusted logistic model. In addition, such an association was not found for self-reported diagnosed hypertension.

On the whole, all associations found in the present study were relatively weak, the highest standardized coefficient being 0.376 between chromium in hair and total proteins. Yet, most of these associations have not resulted in alterations in hematological and biochemical parameters in our population. Whether the alterations found in triglycerides and sodium have been caused by the ingested chromium, remains under investigation. No motor impairment was revealed in the chromium exposed group. To our knowledge, associations between chromium exposure and neurological/neurobehavioral outcomes have not been examined so far. Finally, none self-reported disease was found to be associated with exposure dose in our population. This is also valid for all-sites cancer, a controversial issue in the scientific community.

This work is the first epidemiological study with an individual-based design to investigate health effects associated with long-term consumption of water containing chromium near or above the EU guideline value. In contrast, most of the epidemiological studies are either of ecological type or deal with much higher chromium exposure levels [20,21,22,27]. However, water begins to develop a distinguishable yellow color at concentrations of 0.5–2.0 mg·L^−1^ Cr(VI) and becomes bright yellow (and therefore aesthetically objectionable) at even higher concentrations [7]. The study area is ideal because residents in certain regions have been exposed to geogenic chromium throughout their lifetime. Moreover, additional anthropogenic chromium as a result of industrial activities has been evident in the east region for at least the last 10 years. Considering the limitations of the study, besides that it is a cross-sectional study not allowing causation, there is lack of chromium records in drinking water supplies for a “lifetime period”. This was partly overcome by utilizing the available last-10-year records. Another potential limitation would be that in the estimation of the exposure dose via drinking water, the “usual daily water consumption” captured by the questionnaire was assumed to remain stable over lifetime. However, this assumption seems to be reasonable as it has been reported that the water consumption patterns do not change with age [54].

There is considerable interest in the nature of the oral chromium exposure-response relationship, at low doses [55,56,57,58]. Future research is needed to clarify whether a threshold exists at these doses and consequently to address the need for revision of the current guideline.

## 5. Conclusions

- Associations were found between exposure dose and certain biochemical parameters of the exposed subjects. SRCs ranged from −0.113 for HDL to 0.213 for albumin. Hematocrit and hemoglobin were negatively associated with exposure dose (SPCs −0.094 and −0.093 respectively).
- A positive association was observed between exposure dose and IL-12 levels in blood (SRC 0.308).
- Groups of subjects deviating from normal range for hematological or biochemical parameters were not correlated with exposure dose, except for triglycerides or sodium.
- No motor impairment was revealed in the chromium-exposed group.
- Long-term ingestion of chromium at levels of the same order of magnitude with EU guideline value did not cause deviation from “normal values” in the chromium body burden (blood and hair levels).

## Supplementary Files

Supplementary File 1
