# Supplementary material for: Chromium in Drinking Water: Association with Biomarkers of Exposure and Effect
Source: Int J Environ Res Public Health. 2014 Sep 29;11(10):10125–45. doi: 10.3390/ijerph111010125 (PMC4210971; doi:10.3390/ijerph111010125)
Supplement: Supplementary File 1 [file ijerph-11-10125-s001.pdf]

Chromium in Drinking Water: Association with Biomarkers of Exposure and Effect

Table S1. Classification of the subjects according to their hematological/biochemical values.

Parameter	Normal values	Population proportion (%)		
		Normal	Below normal	Above normal
**Hematological**				
Red Blood Cells (10^{12}/L)	*3.50–5.50*	87.8	0.3	11.9
Hemoglobin (g/dL)	*11.5–16.5*	92.2	4.1	3.7
Hematocrit (%)	*35.0–55.0*	90.8	8.5	0.7
Mean Corpuscular Volume (fL)	*75.0–100.0*	91.5	8.2	0.3
Mean Corpuscular Hemoglobin (pg)	*25.0–35.0*	91.5	8.2	0.3
Mean Cell Hemoglobin Concentration (g/dL)	*31.0–38.0*	99.7	0.3	0.0
Red blood cell Distribution Width (%)	*11.0–16.0*	70.0	0.0	30.0
White Blood Cells (10^9/L)	*3.5–10.0*	92.8	1.4	5.8
Granulocytes percentage (%)	*35.0–80.0*	99.3	0.0	0.7
Lymphocytes percentage (%)	*15.0–50.0*	98.6	0.0	1.4
Mid Cells percentage (%)	*2.0–15.0*	100.0	0.0	0.0
Granulocytes total count (10^9/L)	*1.2–8.0*	98.0	0.0	2.0
Lymphocytes total count (10^9/L)	*0.5–5.0*	99.3	0.0	0.7
Mid Cells total count (10^9/L)	*0.1–1.5*	100.0	0.0	0.0
Platelets (10^9/L)	*100–400*	98.3	0.7	1.0
Plateletcrit (%)	*0.01–9.99*	100.0	0.0	0.0
Mean Platelet Volume (fL)	*9.0–11.0*	42.3	52.9	4.8
Platelet Distribution Width (fL)	*0.1–99.9*	100.0	0.0	0.0
Large Platelet Concentration Ratio (%)	*0.1–99.9*	100.0	0.0	0.0
**Biochemical**				
Glucose (mg/dL)	*76–110*	57.6	21.9	20.5
Urea (mg/dL)	*16.6–50.0*	90.7	7.0	2.3
Creatinine (mg/dL)	*0.50–1.20*	85.4	12.9	1.7
Uric acid (mg/dL)	*2.4–7.0*	89.1	0.3	10.6

Table S1. *Cont.*

Parameter	Normal values	Population proportion (%)		
		Normal	Below normal	Above normal
Triglycerides (mg/dL)	*0–200*	79.8	0.0	20.2
High−Density Lipoprotein (HDL) (mg/dL)	*≥35*	92.1	7.9	0.0
Low−Density Lipoprotein (LDL) (mg/dL)	*0.0–130.0*	57.5	0.0	42.5
Potassium (mmol/L)	*3.50–5.10*	87.7	1.3	11.0
Sodium (mmol/L)	*136.0–145.0*	94.0	6.0	0.0
Alkaline phosphatase (U/L)	*35–129*	94.3	3.7	2.0
Aspartate aminotransferase (sGOT / AST) (U/L)	*0–40*	98.3	0.0	1.7
Alanine aminotransferase (sGPT / ALT) (U/L)	*0–41*	92.7	0.0	7.3
γ−Glutamyl Transpeptidase (γ−GT) (U/L)	*8–61*	73.5	21.9	4.6
Creatine Kinase (U/L)	*26–190*	83.7	0.3	16.0
Lactate Dehydrogenase (U/L)	*135–225*	71.5	24.2	4.3
Amylase (U/L)	*28–100*	93.4	2.0	4.6
Bilirubin total (mg/dL)	*0.2–1.2*	97.3	1.0	1.7
Bilirubin direct (mg/dL)	*0.0–0.3*	97.4	0.0	2.6
Bilirubin indirect (mg/dL)	*0.1–0.7*	98.0	0.0	2.0
Albumin (g/dL)	*3.5–5.2*	83.8	11.6	4.6
Proteins total (g/dL)	*6.0–8.3*	95.0	4.3	0.7
Sfairines (g/dL)	*2.0−3.5*	82.8	6.3	10.9
Calcium (mg/dL)	*8.6–10.2*	84.2	4.7	11.1
Phosphate (mg/dL)	*2.5–4.5*	93.1	3.6	3.3
C−reactive Protein (CRP) (mg/dL)	*0.0–0.5*	89.7	0.0	10.3
**Other outcomes**				
Systolic blood pressure (mm Hg)	*≥140*	67.3	0.0	32.7
Diastolic blood pressure (mm Hg)	*≥90*	73.7	0.0	26.3
Hypertension (mm Hg)	*systolic ≥140 and/or diastolic ≥90*	61.3	0.0	38.7

Table S2. Chemical parameters in drinking water of the study area.

Parameter	Units	Area																				EU guideline value [c]
		A1 [a] (2005–2013)					A2[a] (2004–2008/09) [b]					A2 [a] (2008/09–2013) [b]					A0 [a] (2005–2013)					
		N	Median	Range	25th Perc	75th Perc	*N*	Median	Range	25th Perc	75th Perc	*N*	Median	Range	25th Perc	75th Perc	*N*	Median	Range	25th Perc	75th Perc	
**pH**		*306*	8.0	7.2 −9.2	7.8	8.2	*312*	7.9	6.8–9.1	7.6	8.1	*155*	8.1	7.4–8.5	8.0	8.2	*32*	7.4	7.2–7.8	7.3	7.5	*6.5–9.5*
**Conductivity**	**µmhos cm^{-1}**	*306*	685	573–1269	672	764	*312*	680	75–2200	470	910	*155*	267	243–957	262	291	*32*	552	358–619	545	598	*2500*
**Nitrates**	**mg Ł$^{-1}$ NO_3^-**	*306*	20.7	<0.5–43.6	14.5	24.2	*312*	17.6	<0.5–49.7	13.5	22.7	*155*	6.2	0.5–13.2	5.3	7.0	*32*	22.4	7.5 −24.6	9.7	23.8	*50.0*
**Nitrites**	**mg Ł$^{-1}$ NO_2^-**	*306*	<0.005	<0.005–0.076	<0.005	0.017	*312*	0.016	<0.005–0.122	0.010	0.020	*155*	<0.005	<0.005–0.023	<0.005	<0.005	*32*	<0.005	<0.005–0.008	<0.005	<0.005	*0.500*
**Ammonium**	**mg Ł$^{-1}$ NH_4^+**	*306*	<0.05	<0.05–0.21	<0.05	<0.05	*312*	<0.05	<0.05–0.50	<0.05	0.10	*155*	<0.05	<0.05–0.09	<0.05	<0.05	*32*	0.05	<0.05–0.06	<0.05	0.05	*0.50*
**Sulfates**	**mg Ł$^{-1}$ SO_4^{-2}**	*52*	17	1–150	6	22	*106*	13	1–181	5	30	*36*	24	16–28	22	25	*10*	14	9–20	9	14	*250*
**Chlorides**	**mg Ł$^{-1}$ Cl^-**	*52*	40	4–170	20	56	*106*	60	6–226	40	104	*36*	15	2–21	5	17	*10*	8	6–14	6	14	*250*
**Sodium**	**mg Ł$^{-1}$ Na**	*52*	31	8–70	14	50	*55*	33	4–182	15	61	*36*	6	4–12	4	12	*10*	9	5–13	5	11	*200*
**Fluorides**	**mg Ł$^{-1}$ F^-**	*52*	0.1	<0.1−0.6	<0.1	0.2	*55*	0.1	<0.1–0.2	0.1	0.1	*36*	0.1	<0.1–0.2	0.1	0.1	*10*	0.1	<0.1–0.5	<0.1	0.2	*1.5*
**Boron**	**mg Ł$^{-1}$ B**	*52*	0.1	<0.1−0.6	0.1	0.1	*55*	0.1	<0.1–0.3	0.1	0.2	*36*	0.1	<0.1–0.4	<0.1	0.1	*10*	<0.1	<0.1–0.4	<0.1	0.3	*1.0*
**Cyanides**	**µg Ł$^{-1}$ CN^-**	*52*	<5	<5−10	<5	<5	*55*	<5	<5–15	<5	7	*36*	<5	<5–9	<5	<5	*10*	<5	<5	<5	<5	*50.0*
**Copper**	**mg Ł$^{-1}$ Cu**	*52*	0.002	<0.002–0.011	0.002	0.004	*55*	0.002	<0.002–0.045	0.002	0.004	*36*	0.002	<0.002–0.003	0.002	0.002	*10*	0.003	<0.002–0.008	0.002	0.007	*2.0*
**Chromium total**	**µg Ł$^{-1}$ Cr**	*290*	25.0	0.7–39.9	13.0	28.7	*284*	21.0	<0.5–220	16.2	29.7	*70*	0.5	<0.5–3.2	<0.5	0.7	*32*	1.1	<0.5–3.5	0.7	1.6	*50*
**Chromium hexavalent**	**µg Ł$^{-1}$ Cr(VI)**	*270*	23	<3–38	12	26	*231*	16	<3–196	12	22	*50*	<3	<3	<3	<3	*21*	<3	<3	<3	<3	
**Aluminium**	**µg Ł$^{-1}$ Al**	*52*	<1.5	<1.5–70	<1.5	13.0	*55*	11.5	<1.5–195	3.5	45.0	*36*	118.0	<1.5–200	101.0	164.0	*10*	<1.5	<1.5–20	<1.5	16.0	*200*
**Iron**	**µg Ł$^{-1}$ Fe**	*52*	10	9–175	10	20	*55*	10	1–200	8	11	*36*	20	10–30	10	20	*10*	10	10–10	10	10	*200*
**Manganese**	**µg Ł$^{-1}$ Mn**	*52*	10	<0.5–14	<0.5	10	*55*	10	<0.5–44.0	7	14	*36*	10	<0.5–10.0	<0.5	10	*10*	3	1–6	1	4	*50.0*
**Cadmium**	**µg Ł$^{-1}$ Cd**	*52*	<0.15	<0.15–0.30	<0.15	<0.15	*55*	0.19	<0.15–2.10	<0.15	0.50	*36*	<0.15	<0.15	<0.15	<0.15	*10*	<0.15	<0.15–0.59	<0.15	0.50	*5.0*
**Mercury**	**µg Ł$^{-1}$ Hg**	*52*	<0.3	<0.3–0.4	<0.3	<0.3	*55*	<0.3	<0.3–0.5	<0.3	0.5	*36*	<0.3	<0.3	<0.3	<0.3	*10*	<0.3	<0.3	<0.3	<0.3	*1.0*
**Lead**	**µg Ł$^{-1}$ Pb**	*52*	1.0	<0.5–1.3	<0.5	1.0	*55*	1.0	<0.5–2.5	1.0	2.5	*36*	0.7	<0.5–2.4	<0.5	1.0	*10*	1.0	<0.5–1.9	0.5	1.7	*10.0*
**Nickel**	**µg Ł$^{-1}$ Ni**	*52*	2.5	<1.0−4.6	1.2	3.5	*55*	2.5	<1.0–4.0	2.5	2.5	*36*	<1.0	<1.0–4.0	<1.0	3.1	*10*	1.9	1.8–4.7	1.9	1.9	*20.0*
**Arsenic**	**µg Ł$^{-1}$ As**	*52*	<1.0	<1.0−4.2	<1.0	<1.0	*55*	<1.0	<1.0–2.5	<1.0	1.8	*36*	<1.0	<1.0–3.7	<1.0	1.2	*10*	<1.0	<1.0	<1.0	<1.0	*10.0*
**Selenium**	**µg Ł$^{-1}$ Se**	*52*	<0.4	<0.4	<0.4	<0.4	*55*	2.5	<0.4–2.9	2.5	2.5	*36*	<0.4	<0.4–0.7	<0.4	<0.4	*10*	<0.4	<0.4	<0.4	<0.4	*10.0*
**Antimony**	**µg Ł$^{-1}$ Sb**	*52*	<0.4	<0.4–2.8	<0.4	<0.4	*55*	<0.4	<0.4–2.5	<0.4	2.5	*36*	<0.4	<0.4–2.1	<0.4	0.9	*10*	<0.4	<0.4	<0.4	<0.4	*5.0*
**Trihalomethanes**	**µg Ł$^{-1}$**	*52*	4	<4–−87	<4	23	*55*	<4	<4–9	<4	5	*36*	<4	<4–96	<4	67	*10*	12	<4–30	<4	24	*100*
**Organochlorine pesticides_total**	**µg Ł$^{-1}$**	*52*	<LOD	<LOD	<LOD	<LOD	*55*	<LOD	<LOD	<LOD	<LOD	*36*	<LOD	<LOD	<LOD	<LOD	*10*	<LOD	<LOD	<LOD	<LOD	*0.50*
**Polycyclic Aromatic Hydrocarbons total**	**µg Ł$^{-1}$**	*52*	<LOD	<LOD	<LOD	<LOD	*55*	<LOD	<LOD	<LOD	<LOD	*36*	<LOD	<LOD	<LOD	<LOD	*10*	<LOD	<LOD	<LOD	<LOD	*0.10*

Notes:[a] Area A1: current Cr exposure area, Area A2: past Cr exposure area, Area A0: reference area; [b] 2008 or 2009, depending on the supply change in individual villages; [c] Drinking Water Directive (Council Directive 98/83/EC of 3 November 1998 on the quality of water intended for human consumption).

© 2014 by the authors; licensee MDPI, Basel, Switzerland. This article is an open access article distributed under the terms and conditions of the Creative Commons Attribution license (http://creativecommons.org/licenses/by/4.0/).
